# An accelerometer-derived ballistocardiogram method for detecting heart rate in free-ranging marine mammals

**DOI:** 10.1242/jeb.243872

**Published:** 2022-05-20

**Authors:** Max F. Czapanskiy, Paul J. Ponganis, James A. Fahlbusch, T. L. Schmitt, Jeremy A. Goldbogen

**Affiliations:** 1 Hopkins Marine Station, Department of Biology, Stanford University, Pacific Grove, CA 93950, USA; 2 Scripps Institution of Oceanography, University of California, San Diego, La Jolla, CA 92037, USA; 3 Animal Health Department, SeaWorld of California, San Diego, CA 92109, USA

**Keywords:** Physio-logging, Cetacean, Electrocardiogram, Diving physiology, Cardiovascular physiology, Bio-logging

## Abstract

Physio-logging methods, which use animal-borne devices to record physiological variables, are entering a new era driven by advances in sensor development. However, existing datasets collected with traditional bio-loggers, such as accelerometers, still contain untapped eco-physiological information. Here, we present a computational method for extracting heart rate from high-resolution accelerometer data using a ballistocardiogram. We validated our method with simultaneous accelerometer–electrocardiogram tag deployments in a controlled setting on a killer whale (*Orcinus orca*) and demonstrate the predictions correspond with previously observed cardiovascular patterns in a blue whale (*Balaenoptera musculus*), including the magnitude of apneic bradycardia and increase in heart rate prior to and during ascent. Our ballistocardiogram method may be applied to mine heart rates from previously collected accelerometery data and expand our understanding of comparative cardiovascular physiology.

## Abstract (precis)

**Highlighted Article:** Validation of a computational method for extracting heart rate in free-ranging cetaceans from high-resolution accelerometer data using a ballistocardiogram.

## INTRODUCTION

Recent advances in physio-logging (recording physiological variables using animal-borne devices) have largely been driven by new developments in sensor technology (Hawkes et al., 2021; Williams and Hindle, 2021). For example, new physio-logging tags can detect regional changes in blood flow by incorporating functional near-infrared spectroscopy sensors (McKnight et al., 2021). However, traditional inertial measurement unit (IMU) tags equipped with accelerometers and other inertial sensors can also measure important physiological and related variables, such as wing beat frequency (Patterson et al., 2019) and feeding rate (di Virgilio et al., 2018). Through careful inspection and analysis of high-resolution acceleration, scientists have measured elevated respiration rates following record-breaking dives (Sato et al., 2011), near-continuous feeding by small cetaceans (Wisniewska et al., 2016), social interactions between large cetaceans (Goldbogen et al., 2014), and important biomechanical variables including movement speed (Cade et al., 2018). While physio-logging tags with cutting-edge biomedical technologies push the boundaries of physiological field research, simpler IMU tags have fewer logistical constraints and provide access to more species and larger sample sizes. This is particularly important for species that cannot be restrained or studied in managed care. For example, heart rate has been recorded with an electrocardiogram tag in the wild for just one blue whale (*Balaenoptera musculus*) of the 16 species of baleen whales (Mysticeti) (Goldbogen et al., 2019; but see Ponganis and Kooyman, 1999). Conversely, IMU tags have been deployed on hundreds of individuals of nearly every species in the clade for the last 20 years (Nowacek et al., 2001). These existing datasets (and future IMU tag deployments) could hold additional valuable physiological information, awaiting proper computational methods for mining them.

The ballistocardiogram (BCG) has potential applications to using accelerometers as heart rate monitors in both the wild and managed care (Giovangrandi et al., 2011; Inan et al., 2015; Sadek et al., 2019). Ballistocardiography is a non-invasive method for measuring cardiac function based on the ballistic forces involved in the heart ejecting blood into the major vessels. The BCG originated as a clinical tool in the first half of the 20th century (Starr et al., 1939), but was largely superseded by electrocardiography and echocardiography. However, potential novel applications such as passive monitoring of heart function in at-risk populations (Giovangrandi et al., 2011) has led to a recent resurgence of ballistocardiography research, with advances in hardware (Andreozzi et al., 2021) and signal processing methodology (Sadek et al., 2019). While the BCG is a 3D phenomenon, it is strongest in the cranio-caudal axis (Inan et al., 2015). Along this axis, the waveform is composed of multiple peaks and valleys; most prominent of these is the so-called IJK complex (Pinheiro et al., 2010). The precise physiological mechanism underlying the BCG waveform has not been fully resolved (Kim et al., 2016), but it has been established that the IJK complex occurs during systole and, in humans, occurs at approximately the same time as the T-wave in an electrocardiogram (ECG) (Inan et al., 2015). The BCG J wave is the most robust feature in the waveform and is typically used for detecting heart beats (Inan et al., 2015).

Here, we present a method for generating a BCG from bio-logger cranio-caudal acceleration. We validated our method with a simultaneously recorded ECG on an adult killer whale in managed care (*Orcinus orca*) and applied it to detect heart rate in a blue whale. The relative orientation of a tag on a cetacean's body is often uncertain when bio-loggers are deployed in the wild (Johnson and Tyack, 2003), so isolating acceleration along the cranio-caudal axis is subject to error. Therefore, we also compared a tri-axial BCG with the cranio-caudal BCG. Specifically, we tested three hypotheses to validate our method. First, a cranio-caudal (1D) BCG would, in a controlled setting, produce instantaneous heart rates that are statistically equivalent to ECG instantaneous heart rates. Second, a tri-axial (3D) BCG would, in a field setting, produce a more robust signal than a 1D BCG. Third, BCG-derived heart rate would increase during the later phases of dives, consistent with the progressive increase in heart rate routinely observed prior to and during ascent (Goldbogen et al., 2019; McDonald and Ponganis, 2014).

## MATERIALS AND METHODS

### Animal tagging

#### Killer whale

A 3868 kg adult female killer whale, *Orcinus orca* (Linnaeus 1758), in managed care at SeaWorld of California, San Diego, CA, USA, was double-tagged with an archival Customized Animal Tracking Solutions IMU (CATS, www.cats.is) tag and a custom-built, archival ECG tag on 16 August 2021 as part of clinical animal cardiac evaluations under the SeaWorld USDA APHIS display permit. The ECG tag hardware and data processing procedures were previously described by Bickett et al. (2019). Both tags were deployed by hand and attached with suction cups. We attached the CATS tag on the mid-lateral left chest posterior to the pectoral fin (Movie 1). The CATS tag recorded tri-axial acceleration at 400 Hz, tri-axial magnetometer and tri-axial gyroscope at 50 Hz, pressure at 10 Hz, and video at 30 frames s^−1^. The IMU in the CATS tag was a MPU-9250 (InvenSense, San Jose, CA, USA; www.invensense.com). The accelerometer had dynamic range of ±4 g, sensitivity of 8192 LSB g^−1^ (least significant bit per g) and accuracy of 6.1×10^−6^ g. All sensors were rotated from the tag's frame of reference to that of the whale using MATLAB (MathWorks, Inc., v2020b) tools for processing CATS data (Cade et al., 2021). This rotation aligned the tag's *x*-, *y*- and *z*-axes with the cranio-caudal, lateral and dorso-ventral axes of the whale, respectively. We attached the ECG tag approximately midline on the ventral chest just caudal (posterior) to the axilla and we recorded the ECG at 100 Hz. Individual heart beats in the ECG record were identified from visually verified R-waves using a customized peak detection program (K. Ponganis; Origin 2017, OriginLab Co., Northampton, MA, USA). ECG and IMU were recorded during a spontaneous breath hold while the whale rested at the surface.

#### Blue whale

A 24.5 m blue whale, *Balaenoptera musculus* (Linnaeus 1758), was tagged with an archival, suction-cup CATS IMU tag on 5 September 2018 in Monterey Bay, CA, USA, under permits MBNMS-MULTI-2017-007, NMFS 21678 and Stanford University IACUC 30123 (previously published by Gough et al., 2019). We deployed the tag using a 4 m fiberglass pole from a 6.3 m rigid-hulled inflatable boat and recovered it via radio VHF tracking (as described by Goldbogen et al., 2006). The tag slid behind the left pectoral flipper, similar to the placement of the CATS tag on the killer whale. Tag configuration and data processing followed the same procedure as for the killer whale, including accelerometer specification and sampling rates for inertial sensors and video. The 400 Hz acceleration data were used for ballistocardiography (see ‘Signal processing’, below). We downsampled the multi-sensor data to 10 Hz for movement analysis using the MATLAB CATS tools (Cade et al., 2021).

### Signal processing

The BCG waveform is 3D, but strongest in the cranio-caudal axis (Inan et al., 2015). We tested both 1D (cranio-caudal only) and 3D metrics for identifying heartbeats in acceleration data based on the methods of Lee et al. (2016). For windowed operations (such as moving averages and signal filters), we used 0.5 s windows for killer whale data and 2.0 s windows for blue whale data, corresponding to 200 and 800 data points, respectively. The different window sizes were determined through trial and error to remove noise while retaining signal shape. Generally, longer windows will be necessary for larger animals because of their slower heart rates (Stahl, 1967).

### Procedure

First, we removed noise and de-trended the acceleration signal with a 5th order Butterworth band-pass filter (killer whale: [1–25 Hz], blue whale: [1–10 Hz]) (R package *signal* v0.7-7; https://CRAN.R-project.org/package=signal). The lower cut-off frequency de-trended the data; 1 Hz should be appropriate for most marine mammal species. The upper cut-off frequency removed noise. A suitable upper cut-off frequency will depend on body size; larger species' bodies produce lower magnitude accelerations (Martín López et al., 2021), so more conservative upper cut-off frequencies may be applied to remove more noise without sacrificing signal shape clarity.

Then, we enhanced the IJK complex by differentiating acceleration using a 4th order Savitzky–Golay filter (R package *signal*). Differentiation (i.e. *a_t_*_+1_−*a_t_*, where *a_t_* is the observed acceleration at time step *t*) exaggerates peaks, like the J wave, but it is sensitive to noisy signals. Therefore, additional noise reduction is necessary prior to differentiation. A moving average smoother could remove noise, but it would also reduce the amplitude of peaks. Hence, differentiating Savitzky–Golay filters are preferred in peak-detection algorithms because they remove noise while retaining the general shape of peaks (Samann and Schanze, 2019). We described the resulting signal as ‘differenced acceleration’, rather than jerk, because we did not take the derivative of acceleration with respect to time. The purpose of this signal was to exaggerate a phenomenon in the signal (i.e. the J wave), not to describe a physical quantity (i.e. jerk).

We further enhanced the peaks in the differenced acceleration signal by calculating the Shannon entropy [*H_i_*=−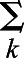
|*a_ik_*|×ln(|*a_ik_*|), where *k* is the acceleration axis]. Additionally, Shannon entropy is strictly positive, which facilitated peak detection. In the 1D BCG, *k* was surge (cranio-caudal acceleration). In the 3D BCG, *k* included surge, sway (lateral acceleration) and heave (dorso-ventral acceleration).

After enhancing the peaks through differentiation and entropy calculation, we removed residual noise by applying a triangular moving average (TMA) smoother. TMAs are equivalent to applying a simple moving average in two passes, which applies greater weight to the middle part of the window and retains peaks and valleys more clearly. After steps 2 and 3, the signal was clear enough that TMAs provided satisfactory results, obviating the need for a more complex algorithm such as a Savitzky–Golay filter at this stage. We described the resulting signal as the BCG.

The BCG contained major peaks (corresponding to heartbeats) and minor peaks (residual noise) (Fig. S1A). We extracted all peaks from the BCG and applied a clustering algorithm to retain major peaks and reject minor peaks. First, we extracted all peaks in the BCG signal using findpeaks() (R package *pracma* v2.3.3; https://CRAN.R-project.org/package=pracma) with a minimum peak distance equivalent to the window size (0.5 s for the killer whale, 2.0 s for the blue whale). For each peak, we calculated its absolute height and its prominence (i.e. height relative to the lowest valley between a peak and its higher neighbors). Then, we calculated each peak's Euclidean distance in height-prominence space from the highest peak (Fig. S1B) and estimated the density distribution of these distances (Fig. S1C). The density distribution was bimodal, with a low-distance peak corresponding to major peaks and a high-distance peak corresponding to minor peaks. We used the distance corresponding to the valley between the two peaks as a threshold for rejecting minor peaks (Fig. S1D).

This procedure may be applied to either 1D (i.e. cranio-caudal only) or 3D acceleration. In the case of 3D acceleration, the band-pass and Savitzky–Golay filters were applied to each axis independently.

### BCG validation with killer whale ECG

We fitted ordinary least squares regression to BCG-derived instantaneous heart rate with respect to ECG-derived heart rate and tested (1) whether the intercept was significantly different from 0 and (2) whether the slope was significantly different from 1. We calculated the mean and standard deviation of absolute error as an equivalence measure (1D BCG only).

### BCG application to blue whale

Dynamic body movements produce an acceleration signal that masks the BCG, so we limited our analyses to motionless periods. These periods occurred during or near the bottom phase of dives between fluke strokes. Strokes were detected from visual examination of the rotational velocity around the lateral axis recorded by gyroscope (*sensu* Gough et al., 2019). We used gyroscopes for stroke detection because (1) they are separate sensors from the accelerometers and (2) strokes are clearly visible in gyroscope signals and are robust to tag placement.

We tested whether the 3D BCG was more robust than the 1D BCG in field data by comparing the signal-to-noise ratios. For both BCGs, we calculated the power spectral density (R package *psd*; Barbour and Parker, 2014). Previously recorded blue whale apneic heart rate was 4–8 beats min^−1^ (Goldbogen et al., 2019), so we quantified signal as the integration of the power spectral density curve from 4 to 8 beats min^−1^ and noise as the integrated remainder, up to 60 beats min^−1^. The sample size recorded by Goldbogen et al. (2019) was one individual, so we could not account for potential inter-individual variation. Nonetheless, 4–8 beats min^−1^ was the best available estimate for typical blue whale apneic heart rate.

We also tested whether BCG-derived instantaneous heart rates were consistent with the range and pattern of heart rates previously observed in the blue whale and other marine mammals; namely, a gradual increase in heart rate later in the dive, especially during the final ascent (Goldbogen et al., 2019; McDonald and Ponganis, 2014). We assigned dive start and end times when the whale swam deeper than 2 m, retaining dives that exceeded 10 m depth and 5 min duration. Dive times were normalized from 0 (start of dive) to 1 (end of dive). We regressed instantaneous heart rate against normalized dive time using robust Theil–Sen regression (to account for heteroscedascity) (R package *RobustLinearReg* v1.2.0; https://CRAN.R-project.org/package=RobustLinearReg; Sen, 1968; Theil, 1992) and tested whether the slope was greater than 0.

### Reproducibility

The data and code used in this analysis were packaged as a research compendium, containing the data, code and an executable version of the manuscript. We used the R package *rrtools* (https://github.com/benmarwick/rrtools v0.1.5; Marwick et al., 2018) to initialize the compendium, which was written as an R package. This approach promotes reproducibility and facilitates adoption by other researchers (Alston and Rick, 2021; Powers and Hampton, 2019; Stodden et al., 2018). The steps described in ‘Signal processing’ (above) were implemented as functions in the R package, and the executable manuscript demonstrates how to use those functions to perform the analyses presented in this study.

## RESULTS AND DISCUSSION

### BCG validation with killer whale ECG

**Fig. 1.**
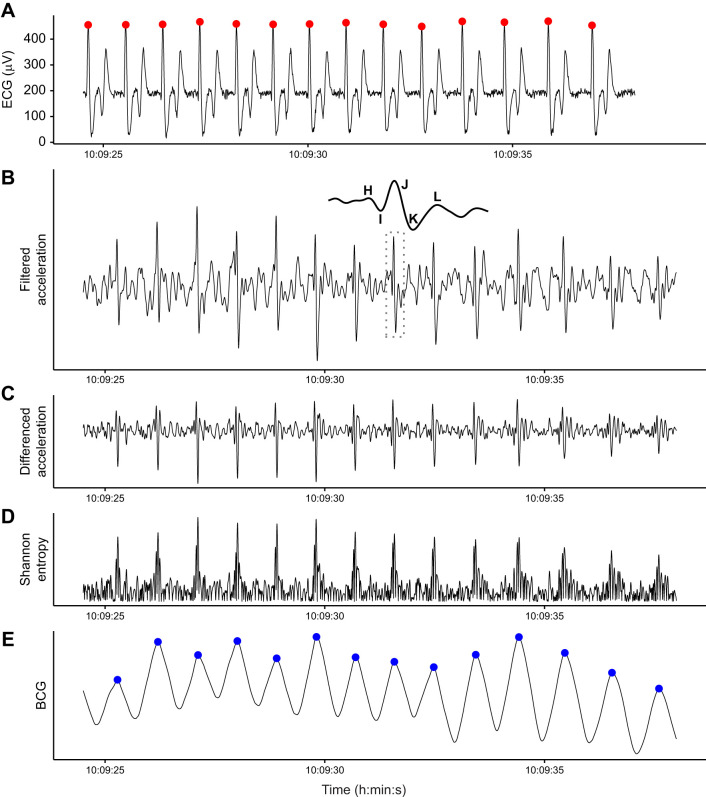
**Ballistocardiogram (BCG) validation.** (A,E) The electrocardiogram (ECG, recorded by ECG tag; A) and 1D BCG (processed from the cranio-caudal acceleration recorded by the IMU tag; E) produced nearly identical heart beat predictions for the killer whale. (B–D) Intermediate steps in the BCG signal processing procedure. (B) Cranio-caudal axis acceleration after band-pass filtering. Inset shows the IJK complex with surrounding H and L waves for the region bounded by the dashed box. (C) Peaks enhanced after forward differencing acceleration (see Materials and Methods ‘Procedure’). (D) A strictly positive signal after calculating Shannon entropy. The *y*-axis scale units were excluded because filtering introduces magnitude distortion and only the relative shape of the signal is relevant to the analysis.

The ECG and BCG yielded nearly identical heart rate estimations (Fig. 1). We collected 14 s of simultaneous ECG and BCG data during a motionless breath hold at the surface. Logistical constraints prevented us from gathering a longer sample, as these data were collected secondary to other projects. BCG-derived instantaneous heart rates were within 0.8±0.5% of the ECG-derived rates (mean±s.d.). Ordinary least squares regression of BCG heart rates on ECG heart rates yielded a slope of 1.02±0.04 and intercept of −1.62±2.71 (mean±s.e.), which were not significantly different from the hypothesized 1 and 0, respectively (Fig. 3C).

### BCG application to blue whale

**Fig. 2.**
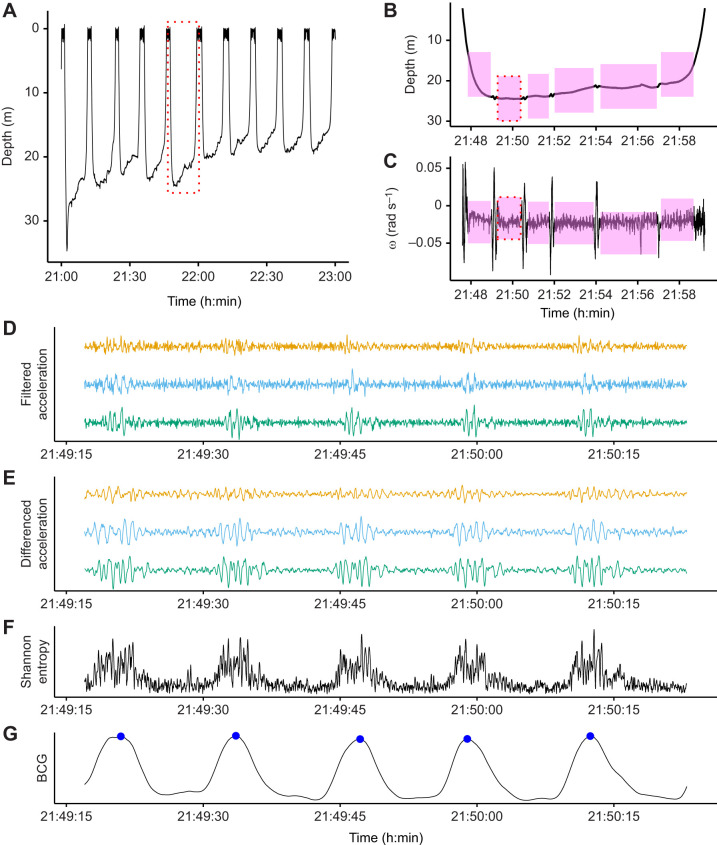
**A 2** **h sample of resting blue whale data.** (A) The depth profile consisted of 10 dives to 20–30 m. The red dashed box indicates the dive expanded in B and C. (B,C) Depth (B) and rotational velocity (ω; C) around the lateral axis for a single dive. Rotational velocity was used to identify motionless periods (pink). Red dashed boxes indicate the motionless period in D–G. (D) Band-pass filtered triaxial acceleration, with cranio-caudal in orange, lateral in blue and dorso-ventral in green. (E) Peaks enhanced after forward differencing acceleration (see Materials and Methods ‘Procedure’). (F) The Shannon entropy combines information from all three axes and makes the signal strictly positive. (G) Smoothing the Shannon entropy facilitates robust peak detection. Detected heart beats are in blue. The *y*-axis scale units were excluded in D–G because the filtering process introduces magnitude distortion and only the relative shape of the signal is relevant to the analysis.

We generated 1D and 3D BCGs for 2 h of data, including 10 rest dives and 51 motionless periods totaling 76.9 min (64.1% of the 2 h record) (Fig. 2A–C).

**Fig. 3.**
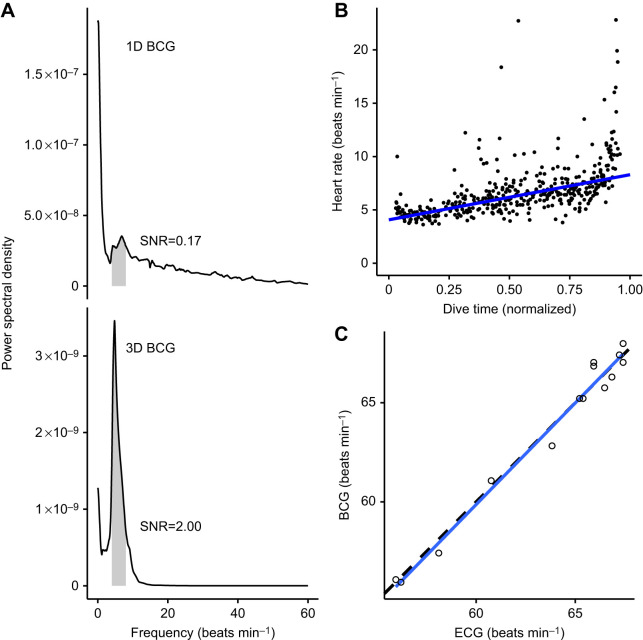
**1D and 3D BCG validation.** (A) The blue whale signal-to-noise ratio (SNR) was higher for the 3D BCG (lower panel) than for the 1D BCG (cranio-caudal acceleration only; upper panel). Each panel shows the power spectral density for the BCG. Based on previously observed blue whale heart rates, 4–8 beats min^−1^ was considered signal (gray shading). The SNR was calculated as the ratio of the area under the curve in the signal band to the area under the rest of the curve, up to 60 beats min^−1^. (B) Heart rates observed in the 3D blue whale BCG followed characteristic diving physiology patterns. Heart rate is lowest at the start of the dive (∼4–5 beats min^−1^), increasing towards ascent (∼8–9 beats min^−1^). Points indicate instantaneous heart rate and the line is a Theil–Sen regression. Outliers likely represent premature beats which are common in heart rate profiles during dives of cetaceans, pinnipeds and penguins (Andrews et al., 1997; Goldbogen et al., 2019; McDonald and Ponganis, 2014; Wright et al., 2014). (C) BCG- and ECG-derived instantaneous heart rates were equivalent in the killer whale. The slope and intercept of the ordinary least squares regression of BCG- on ECG-derived instantaneous heart rates (solid blue line) were not significantly different from 1 and 0, respectively (dashed black line).

The 3D BCG (Fig. 2) produced a more robust signal (i.e. higher signal-to-noise ratio) than the 1D BCG, which used only cranio-caudal acceleration (Fig. 3A). The signal-to-noise ratio was 2.00 for the 3D BCG, compared with 0.17 for the 1D BCG. Although the power spectral density curve for the 1D BCG had a peak in the 4–8 beats min^−1^ frequency range, most of the signal's power was concentrated in lower frequencies. Conversely, the 3D BCG's power was concentrated precisely in the 4–8 beats min^−1^ frequency range, with only a smaller peak in the lower frequencies.

The 3D BCG exhibited increasing heart rate over the course of dives. Average heart rate increased from 4.1 beats min^−1^ at the start of dives to 8.3 beats min^−1^ at the end of dives (Theil–Sen regression, *P*<10^−10^) (Fig. 3B).

### Limitations and considerations for future applications

While the BCG method presented here holds the potential to mine existing and future marine mammal bio-logging datasets for information about cardiovascular function, it has several limitations compared with ECG methods. Most importantly, BCGs are highly sensitive to movement artifacts (Inan et al., 2015), so only motionless periods are valid for analysis. This limits the behavioral and physiological contexts in which heart rate may be measured. For example, the BCG is probably an inappropriate method for quantifying the magnitude of surface tachycardia (Goldbogen et al., 2019) and exercise modulation of bradycardia (Noren et al., 2012), because of movement artifacts during those activities. Additionally, we did not test whether the BCG is robust to tag placement location. The blue whale data presented in this study were collected when a dorsally deployed tag slipped to the lateral chest cavity behind a flipper, where it is reasonable to expect greater accelerations caused by heart beats than from a tag farther from the animal's center of mass. It is possible that the ballistic forces generated by heart beats are strong enough to produce an interpretable BCG for a variety of potential tag deployment locations, but this likely varies with animal body size, as well as accelerometer sampling rate and sensitivity.

When auditing existing bio-logging data and planning future tag deployments for BCG analysis, careful consideration should be paid to sampling rate. As a rule of thumb in signal processing, the sampling rate should be at least twice the frequency of the phenomenon of interest. In the case of the BCG, the relevant frequency is that of the BCG waveform, not the heart rate. In humans, the power of the IJK complex (the part of the BCG waveform used for heart beat detection) is concentrated between 4 and 7 Hz (Moukadem et al., 2018). It is unlikely that marine mammal BCG waveforms have a higher frequency than those of humans, owing to their generally larger body sizes. Therefore, it is possible that BCGs may be generated for accelerometer sampling rates as low as 10–15 Hz. Conservatively, the authors recommend a sampling rate of no less than 50 Hz (i.e. twice the upper cut-off frequency of the widest bandpass filter used in this study).

Future bio-logging BCG methodology research should address the limitations imposed by tag placement and movement artifacts. We used accelerometers in this study because of their prevalence in bio-logging research, but it is possible that other widely used bio-logger sensors, such as gyroscopes and/or magnetometers, could produce a clearer signal in a greater variety of contexts. Alternative bio-logger housing designs, such as limpet-style tags (Andrews et al., 2008) or ‘marine skin’ (Nassar et al., 2018), could reduce noise, boost the signal-to-noise ratio and make the method more widely applicable.

### Conclusions

Here, we present a BCG method for detecting resting apneic heart rate in cetaceans using accelerometers. We validated the method in a controlled setting with simultaneous ECG and in a field setting by confirming expected physiological patterns. As accelerometer tags have been deployed on many cetacean species for multiple decades, this method may be applied to mine existing datasets and better understand how heart rate scales with body size and other biological factors. It may also provide additional data for conservation physiology applications. For example, BCGs extracted from gliding phases before and after controlled sonar exposure experiments could quantify the physiological response to anthropogenic disturbance (Southall et al., 2019). Even as the field of physio-logging progresses with new hardware innovations (Fahlman et al., 2021; Hawkes et al., 2021; Williams and Hindle, 2021), this method demonstrates that computational advances can derive new insights from traditional sensors.

## Supplementary Material

Supplementary information
