# Supplementary material for: An accelerometer-derived ballistocardiogram method for detecting heart rate in free-ranging marine mammals
Source: J Exp Biol. 2022 May 20;225(10):jeb243872. doi: 10.1242/jeb.243872 (PMC9167577; doi:10.1242/jeb.243872)
Supplement: Supplementary information [file jexbio-225-243872-s1.pdf]

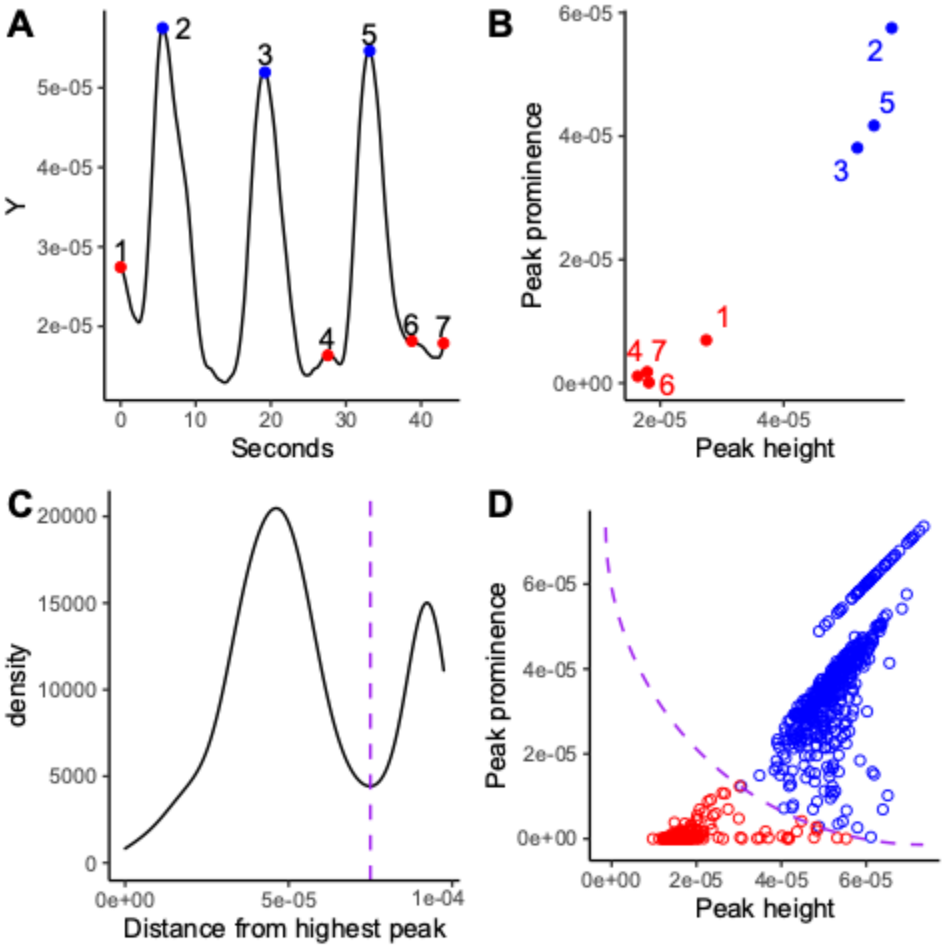

Fig. S1. Heuristic method for discriminating between major and minor peaks. **A:** Minor peaks (red points) in the ballistocardiogram (BCG, line) were not considered heart beats. Only major peaks (blue points) were retained for analysis. The BCG for one motionless period shown here. **B:** We used peak height and prominence (i.e. height relative to the contour surrounding a higher peak) to heuristically differentiate major and minor peaks. For each peak, we calculated the Euclidean distance (in height-prominence space) to the highest peak overall. The peaks in **A** shown here in height-prominence space. **C:** The distance to the highest peak exhibited a bimodal distribution. We chose a distance threshold (dashed purple line) corresponding to the valley in the density curve. **D:** All peaks found in the BCG across all motionless periods in height-prominence space. Solid and hollow points as in **A**. The dashed purple curve corresponds to the distance threshold in **C**.

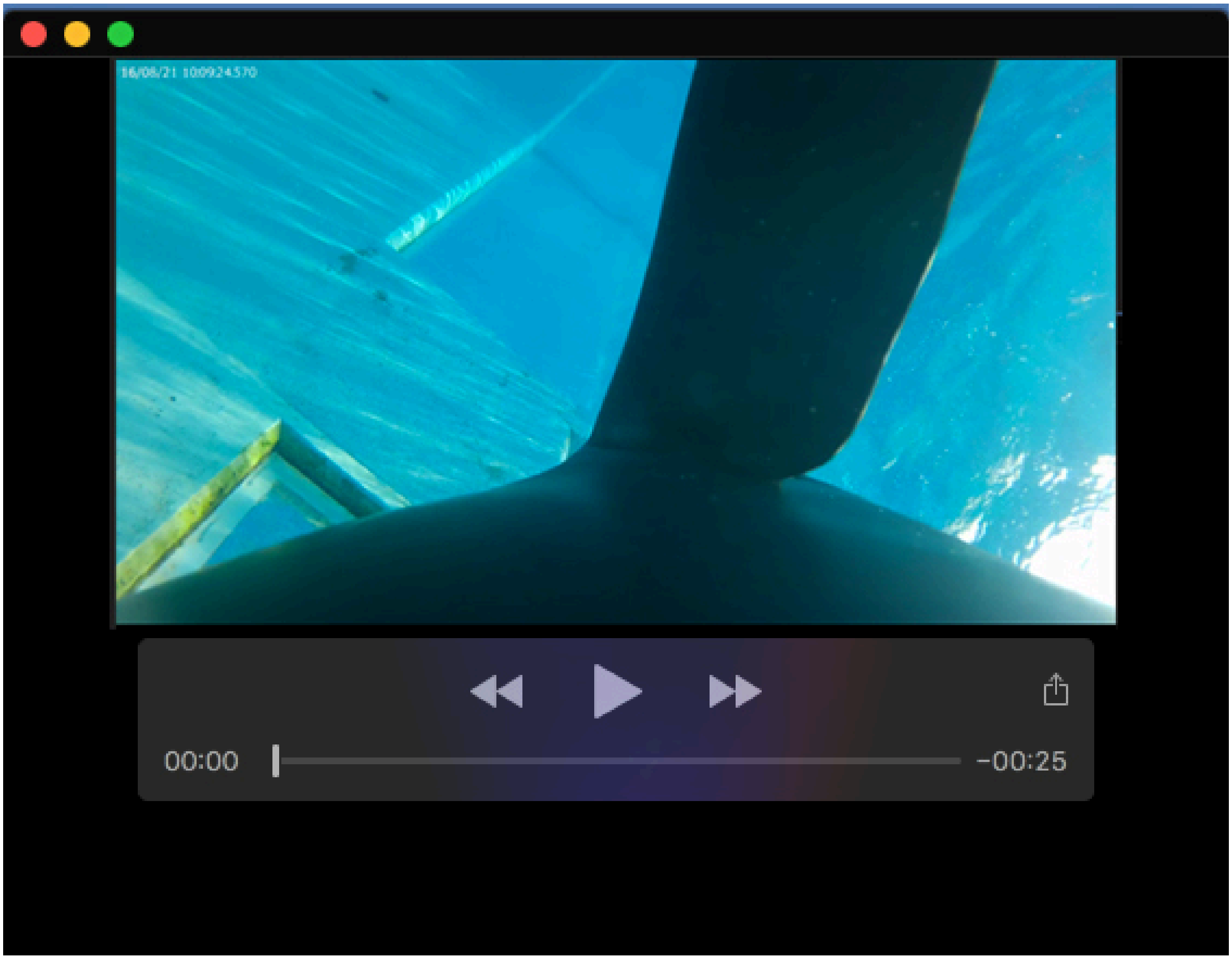

Movie 1. Tag placement on killer whale. Video from the CATS tag shows the placement of the tag on the killer whale's mid-lateral chest, posterior to the left pectoral flipper.
